# EGFP-EGF1-Conjugated PLGA Nanoparticles for Targeted Delivery of siRNA into Injured Brain Microvascular Endothelial Cells for Efficient RNA Interference

**DOI:** 10.1371/journal.pone.0060860

**Published:** 2013-04-10

**Authors:** Chen Chen, Heng Mei, Wei Shi, Jun Deng, Bo Zhang, Tao Guo, Huafang Wang, Yu Hu

**Affiliations:** 1 Institute of Hematology, Union Hospital, Tongji Medical College, Huazhong University of Science and Technology, Wuhan, Hubei, PR China; 2 Targeted Biotherapy Key Laboratory of Ministry of Education, Wuhan, Hubei, PR China; Universidad de Castilla-La Mancha, Spain

## Abstract

Injured endothelium is an important target for drug and/or gene therapy because brain microvascular endothelial cells (BMECs) play critical roles in various pathophysiological conditions. RNA-mediated gene silencing presents a new therapeutic approach for treating such diseases, but major challenge is to ensure minimal toxicity and target delivery of siRNA to injured BMECs. Injured BMECs overexpress tissue factor (TF), which the fusion protein EGFP-EGF1 could be targeted to. In this study, TNF alpha (TNF-α) was chosen as a stimulus for primary BMECs to produce injured endothelium *in vitro*. The EGFP-EGF1-PLGA nanoparticles (ENPs) with loaded TF-siRNA were used as a new carrier for targeted delivery to the injured BMECs. The nanoparticles then produced intracellular RNA interference against TF. We compared ENP-based transfections with NP-mediated transfections, and our studies show that the ENP-based transfections result in a more efficient downregulation of TF. Our findings also show that the TF siRNA-loaded ENPs had minimal toxicity, with almost 96% of the cells viable 24 h after transfection while Lipofectamine-based transfections resulted in only 75% of the cells. Therefore, ENP-based transfection could be used for efficient siRNA transfection to injured BMECs and for efficient RNA interference (RNAi). This transfection could serve as a potential treatment for diseases, such as stroke, atherosclerosis and cancer.

## Introduction

Endothelial cells lining the inner surfaces of microvessels form a barrier that actively participates in the blood–tissue exchange of plasma fluid, proteins and cells. Thus these cells play an important role in maintaining circulatory homeostasis and the physiological functions of different organs. Brain microvascular endothelial cells (BMECs) are a type of endothelial cell and act as a barrier to any invading pathogens [1], [2] in the brain. Injured endothelial cells are associated with crucial events that occur during the development of a variety of diseases, such as stroke, atherosclerosis, and tumor [3]; thus, the targeting of these cells could be a treatment for these diseases.

Tissue factor (TF) is a plasma membrane glycoprotein and the primary initiator of blood coagulation. TF is involved in the thrombosis and inﬂammation that are associated with sepsis, atherosclerosis, and cancer. TF can participate in other pathological processes, such as metastasis, tumor-associated angiogenesis, and tumor growth [4], [5]. The therapeutic targeting of TF is a promising method for treating these diseases. Under normal conditions, the endothelium provides an anticoagulant surface, a property that is lost after injury. Following vessel injury, the membrane-bound TF is exposed to circulating blood, which activates coagulation factor VII (FVII) and may trigger thrombosis [6], [7]. Numerous inflammatory mediators such as TNF-α, interleukin-1, bacterial lipopolysaccharide, and thrombin induce endothelial TF expression by injuring the endothelium [8], [9]. Thus, TNF-α can act as a stimulus by increasing the TF expression in endothelial cells.

Small interfering RNA (siRNA) is a promising strategy in gene therapy for the treatment of diverse diseases because of its superior capability for silencing a target gene [10] using an RNA-induced silencing complex (RISC) [11]. However, because of its size and negative charge, siRNA cannot easily cross cell membranes. Delivery has therefore been one of the most significant challenges in siRNA therapeutics [12], [13].

Currently, nanoparticles composed of PLGA are attractive for use in gene silencing applications because of their high stability, low toxicity, and the possibility for controlled release of their cargo [14]–[16]. Moreover, targeting has been achieved by attaching a targeting molecule or protein to these nanoparticles [17]. Our previous studies indicated that the fusion protein derived from FVII has a specific TF binding capacity but dose not cause coagulation [18] and can enhance the binding ability of PLGA nanoparticles to regions with exposed TF [19]. Thus, EGFP-EGF1 modified PLGA nanoparticles may be a suitable delivery vehicle for siRNAs.

**Table 1:** The particle size and zeta potential of NP and ENP with siRNA-loaded or non-loaded.

nanoparticles	Mean size(nm)	Zeta potential(mV)
ENP	100.06±5.12	−12.31±2.01
NP	92.86±2.12	−11.71±3.98
siRNA/ENP	106.08±3.23	−11.15±3.19
siRNA/NP	96.59±4.38	−9.11±0.98

Measured in double-distilled water n = 3, mean±SD.

In this study, TF was chosen as the therapeutic target for siRNA therapy. The endothelial cells that are injured by TNF-α overexpressed TF. The nanoparticles were composed of the biodegradable and biocompatible polymer poly-(lactic-co-glycolic acid) (PLGA) and the nanoparticles and the EGFP-EGF1 fusion protein was attached to the nanoparticle surfaces. The EGFP-EGF1-conjugated PLGA nanoparticles (ENPs) were used as a new targeted carrier for TF-specific siRNA and enabled siRNA delivery into injured primary BMECs. The physical properties of the nanoparticles, the cytotoxicity of the nanoparticles, the siRNA release *in vitro* from the nanoparticles, and the gene silencing effect were determined. To our knowledge, this is the first time that the nanoparticle targeted delivery of TF-specific siRNA to injured primary endothelial cells has been successfully studied.

## Materials and Methods

### 2.1 Materials and Animals

The *E. coli* strain BL21 (DE3) and plasmid pET-28a-EGFP-EGF1 were maintained in our laboratory. Poly-(D, L lactic-co-glycolic acid) (PLGA, 50∶50, inherent viscosity of 0.89, MW∼100 kDa) was purchased from Absorbable Polymers (USA). Methoxy-poly-(ethylene glycol) (M-PEG, MW 3000 Da) was purchased from the NOF Co. (lot no.14530, Japan) and Maleimide-PEG (Mal-PEG, MW 3400 Da) was purchased from Nektar Co. (lot no.PT-08D-16, USA). Cy3-labeled negative siRNAs were purchased from the RiboBio Co. (Guangzhou, China). Rabbit polyclonal antibody against to the rat TF antibody was purchase from Santa Cruz Biotechnology (USA). Flow cytometry antibody (CD142) was purchased from BD Biosciences (USA). Medium 131, MVGS, Dulbecco’s Modified Eagle’s Medium (high glucose) (DMEM), and fetal bovine serum (FBS) were purchased from Life Technologies Corporation (USA). All other chemicals were analytical reagent grades, purchased from the Sinopharm Chemical Reagent Co. (China).

**Table 2:** The EE and DLC of siRNA-loaded NP and ENP.

nanoparticles	siRNA/ENP	siRNA/NP
Drug entrappingefficiency (%)	81.33±0.24	79.05±0.43
Drug loading capability(µg/mg)	1.36±0.01	1.32±0.01

Sprague Dawley (SD) rats (50–60 g, ♂) were provided by the Center of Experimental Animals at Tongji Medical College (Wuhan, China). The protocols for treating the animals during the experiment were evaluated and approved by the Tongji Medical College ethical committee.

### 2.2. Cells

BMECs were separated from Sprague Dawley (SD) rats (50–60 g, ♂) as previously described [20]–[22] and cultured in Medium131 (M131), which has been supplemented with 5% MVGS, at 37°C in a humidified atmosphere of 5% carbon dioxide(CO_2_). The cells were cultured in the medium and the experiments were conducted in 3–6 passages. TNF-α [23] was added, 100 ng/ml, to injure the endothelium *in vitro*. Rat C6 glioma cells (Chinese Academy of Medical Sciences and Peking Union Medical College, Beijing, China) were cultured in DMEM (high glucose) which consisted of 10% FBS and antibiotics (including 100 µg/ml of penicillin, and 100 µg/ml of streptomycin) at 37°C in a humidified atmosphere with 5% CO_2_.

### 2.3. Synthesis and *in vitro* Screening of siRNAs

The three siRNAs with the lowest predicted off-target potentials and 100% homology with the rat TF gene sequence NM_013057.2 were selected for synthesis and screening. The siRNAs were obtained from the Life Technologies Corporation (USA).

Rat C6 glioma cells, which naturally overexpress TF, were transfected with siRNAs using the Lipofectamine 2000 reagent (Invitrogen, USA) according to the manufacturer’s protocols in concentration ranging from 10 nM to 100 nM. TF mRNA levels were quantified 24 h after transfection by real time PCR and normalized using GAPDH mRNA. The best RNAi concentration was 40 nM. The best duplex, with the sequence of 5′-GCAAUGACUUGGGUUAUAUdTdT-3′ (sense) and, 5′-AUAUAACCCAAGUCAUUGCdTdT-3′ (antisense), was selected for scaling up, formulation and subsequent *in vivo* work.

### 2.4. Preparation of siRNA-loaded NPs and siRNA-loaded ENPs

The siRNA-loaded NPs were prepared using a water-in-oil-in-water (w/o/w) double emulsion solvent evaporation method similar to the one previously reported [24]. In brief, a solution of 40 µg of siRNA in 50 µl of DEPC MilliQ water containing Ac-BSA [25] was mixed with dichloromethane (DCM) containing Me-PEG-PLGA and Mal-PEG-PLGA (weight ratio 10∶1), and the mixture was emulsified by sonicating it for 20 s in a primary w/o emulsion. Two milliliters of 2% sodium cholate in MilliQ water was poured directly into the primary emulsion, and this mixture was further emulsified by sonicating it 20 times (1 s sonication and 1 s arrest) to form a w/o/w double emulsion. The resulting emulsion was diluted with 35 ml of 2% sodium cholate in MilliQ water and heated for 15 min at 37°C to evaporate the DCM. The nanoparticles were then collected using ultracentrifugation at 14000 rpm for 40 min at 4°C and resuspended in DEPC-treated PBS (0.01 M, pH = 7.4). EGFP-EGF1-PLGA nanoparticles were prepared by incubating purified thiolated EGFP-EGF1 with the PLGA nanoparticle solution for 8 h under an N_2_ gas atmosphere. The siRNA-loaded ENPs were passed through a 1.5×20 cm Sepharose CL-4B column and eluted using PBS (0.01 M, pH = 7.4) to remove the unconjugated proteins. The nanoparticles were then collected using ultracentrifugation at 14000 rpm for 40 min at 4°C and resuspended in DEPC-treated PBS (0.01 M, pH = 7.4). The preparation of NPs labeled with 6-coumarin was the same as above except that 30 µl of 6-coumarin (1 mg/ml stock solution in methyl cyanides) was added into the 1 ml of DCM before primary emulsification.

### 2.5. Characterization of Nanoparticles

The mean diameter and zeta potential of the nanoparticles were determined by dynamic light scattering (DLS) using the zeta potential/particle sizer Nicomp 380 ZLS (Particle Sizing Systems, Santa Barbara, USA). The nanoparticles were morphologically examined by transmission electron microscopy (H-600, Hitachi, Japan). *In vitro* release experiments were performed at 37°C in PBS (0.01 M) with pH = 7.4 and pH = 4.0 for a period of 72 h. The siRNA-loaded ENPs were incubated at a nanoparticle concentration of 10 mg/mL in a rotary shaker at 100 rpm and 37°C. Three samples were taken for each time point studied. The samples were treated using the same method described by Dongmei et al. [14]. The extracted siRNAs were quantified by nucleic acid determination (NanoDrop1000 Spectrophotometer,Thermo Scientific, USA). Each sample was analyzed three times. The drug loading capacity (DLC) and encapsulation efficiency (EE) were determined using the same method and calculated according to the following equations:
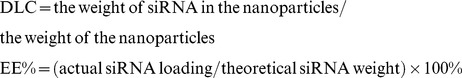



### 2.6. Cellular Uptake and siRNA Tracking Study

BMECs were seeded at a density of 1×10^5^ per well in 6-well plates, incubated for 24 h, and checked under a microscope for similar confluency and morphology. The ENPs and NPs were labeled with 6-coumarin. The BMECs were treated with TNF-α (100 ng/ml). Simultaneously, one type of nanoparticle was added in the presence of serum-free media, and the mixture was incubated for 4 h at 37°C. The cells were washed 2 times with PBS (0.01 M, pH = 7.4) and immobilized with 4% paraformaldehyde for 20 min at room temperature. To stain the cell nuclei, the cells were incubated with DAPI (1 µg/ml) for 10 min at room temperature avoid light. Normal BMECs were incubated with the nanoparticles or PBS to serve as a control. To visualize the siRNA intracellular distribution, double-stranded siRNA was labeled with Cy3.

### 2.7. In Vitro Transfection Experiments

The BMECs were seeded at a density of 1×10^5^ per well in 6-well plates and grown to reach 70–80% conﬂuence. The fresh serum-free DMEM (high glucose) containing different nanoparticle formulations, including NPs, ENPs, siRNA-loaded NPs, and siRNA-loaded ENPs, and TNF-α (100 ng/ml) was mixed and incubated at 37°C in a 5% CO_2_ humidified atmosphere for 6 h. The final concentration of siRNA in these nanoparticles was 40 nM. A negative control was prepared with PBS alone. After treatment, the cells were washed 2 times with PBS, trypsinized by 0.25% trypsinase, and collected. The TF expression was determined by the real time PCR, western blot, and ﬂow cytometry. Moreover, cell supernatants were harvested for TF activity assays.

#### 2.7.1. Real time PCR

To determine the downregulation of the TF mRNA level, total RNA was extracted using TRIzol reagent (Invitrogen, USA). Conversion of total cellular RNA to cDNA was conducted using the Prime Script RT reagent Kit (Takara Bio, Japan). The total cDNA pool was obtained and served as a template for subsequent PCR amplification with primers specific to TF (sense primer: 5′-GTGCACTGAGCAATGGAAGA-3′, antisense primer: 5′-AGGCCATGAAGGGAGTCTTT-3′) and to GAPDH (sense primer: 5′- ATGGTGGTGAAGACGCCAGTA-3′, antisense primer: 5′-GGCACAGTCAAGGGCTGAGAATG-3′). Quantitative PCR amplification was performed in a real-time fluorescent measurement system (ABI Step One, USA) using the SYBR Premix Ex Taq™ kit (Takara Bio, Japan), according to the manufacturer’s protocols. The TF mRNA levels were normalized using GAPDH.

#### 2.7.2. Western blot assay

To determine the TF protein levels, the collected BMECs were washed twice with ice-cold PBS (0.01 M, pH = 7.4) and then lysed in a protein buffer containing 1∶50 protease inhibitor cocktails (Guge Biotech Co., China). Cell lysates were centrifuged for 20 min at 12000 rpm and 4°C (Eppendorf Centrifuge5804R, USA) and the supernatant was collected. Total protein extracts were determined using the BCA Protein Assay Kit (Thermo Scientific, USA). Equal amounts of protein (30 µg) were separated via SDS polyacrylamide gel electrophoresis, electrotransferred to polyvinylidene fluoride (PVDF) membranes, and exposed to a polyclonal anti-TF antibody (1∶250, Santa Cruz, USA). Immunoreactive bands were detected by chemiluminescence using an ECL detection kit (Thermo Scientific, USA) with GAPDH (1∶1000, Boster, China) as a loading control. The optical intensities of the TF bands were normalized to the GAPDH protein bands using the Gel-Pro Analyzer software.

#### 2.7.3. Flow cytometry

To confirm the western blot results, the collected cells were washed 2 times with cold PBS (0.01 M, pH = 7.4). Viable cells were counted and resuspended in 100 µl of PBS (10^6^/cell). The cells were stained with 10 µl of a PE-labeled anti-CD142 antibody (BD Biosciences, USA) and kept on ice for 30 min in the dark, and one sample was stained with 3 µl of PI as a control. The samples were then washed, resuspended (500 µl of PBS), and analyzed using a flow cytometery (Becton Dickinson FACSort, USA) to determine the TF protein levels.

#### 2.7.4. TF activity assay

The TF activity of different treatments was determined using the AssaySense Human Tissue Factor Chromogenic Activity Assay Kit (AssayPro, USA). The cell supernatants were incubated at 37°C with human FVIIa and FX, enabling the formation of TF/FVIIa complexes. The conversion of FX to FXa was measured by using the ability of FXa to cleave a chromogenic substrate. The absorbance (A) was measured at 405 nm with microplate reader. A standard curve was established using recombinant human TF lipoprotein to ensure that the results were in the linear range of detection.

### 2.8 Cytotoxicity Assay

BMECs were seeded in 24-well plates at a density of 2.5×10^4^ cells per well in 500 µl of M131 and incubated for 24 h. The cells were then transfected, as described earlier, using Lipofectamine 2000 or nanoparticles. There were four wells for each mixture. Twenty four hours following transfection, 40 µl of CCK-8 (Dojindo, Japan) was added to each well, and the mixtures were incubated for 4 h. The absorbance (A) was measured at 450 nm with a microplate reader (BioTek, USA). The cell viabilities were normalized using blank cells.

To evaluate the cytotoxicity of ENPs at higher concentrations, BMECs were seeded in 96-well plates at a density of 5000 cells per well in 100 µl of M131 and incubated for 24 h. After the media was replaced with a fresh medium, ENPs with siRNA concentrations ranging from 0 mg/ml to 4 mg/ml were added into the cells, followed by a 24 h incubation period. There were four cells for each concentration. CCK-8 assays were performed similarly to the experiments above. NPs with siRNA, ENPs, and siRNAs were used as controls.

## Results

### 3.1. Characterization of Nanoparticles

The nanoparticles prepared by blending M-PEG-PLGA and Mal-PEG-PLGA had an average diameter of approximately 92 nm, and this diameter increased to approximately 100 nm after EGFP-EGF1 conjugation. When the siRNAs were entrapped in the nanoparticles, the ENPs and NPs had average diameters of 106 nm and 96 nm, respectively. The zeta potential values of siRNA-loaded NPs and siRNA-loaded ENPs were negative and ranged from −9 mV to −11 mV (Table. 1.).

**Figure 1:**
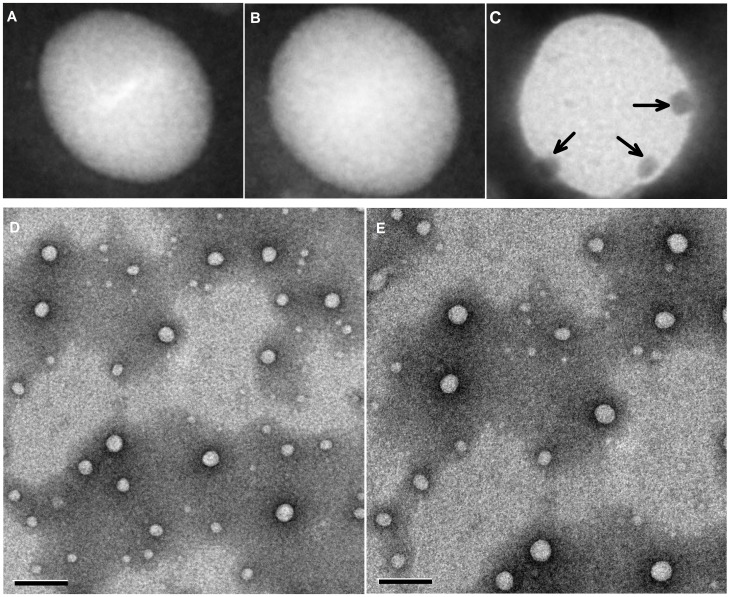
The different nanoparticles that were negatively stained with a 1% phosphotungstic acid solution. (A) siRNA-loaded NPs stained with EGF primary antibody and with 10 nM of colloidal gold-labeled rabbit anti-goat IgG. (B) siRNA-loaded ENPs stained with 10 nM of colloidal gold-labeled rabbit anti-goat IgG. (C) siRNA-loaded ENPs stained with EGF primary antibody and stained with 10 nM of colloidal gold-labeled rabbit anti-goat IgG. The arrows point to the fusion protein EGF1, which conjugated to the surfaces of the nanoparticles. (D) siRNA-loaded NPs. (E) siRNA-loaded ENPs. The bars shown in (D) and (E) are 200 nm.

The nanoparticles were generally spherical and uniform. And the conjugation of the EGFP-EGF1 fusion protein is shown in Fig. 1.C. For the PLGA nanoparticles with a high encapsulation efficiency prepared using the double emulsion solvent evaporation (DESE) method [26], the drug loading capacity of the ENPs and NPs were 1.36±0.01 µg/mg and 1.32±0.01 µg/mg, respectively. No differences were observed in the drug loading capacity (DLC) and encapsulation efficiency (EE) of ENPs and NPs (Table. 2.).

**Figure 2:**
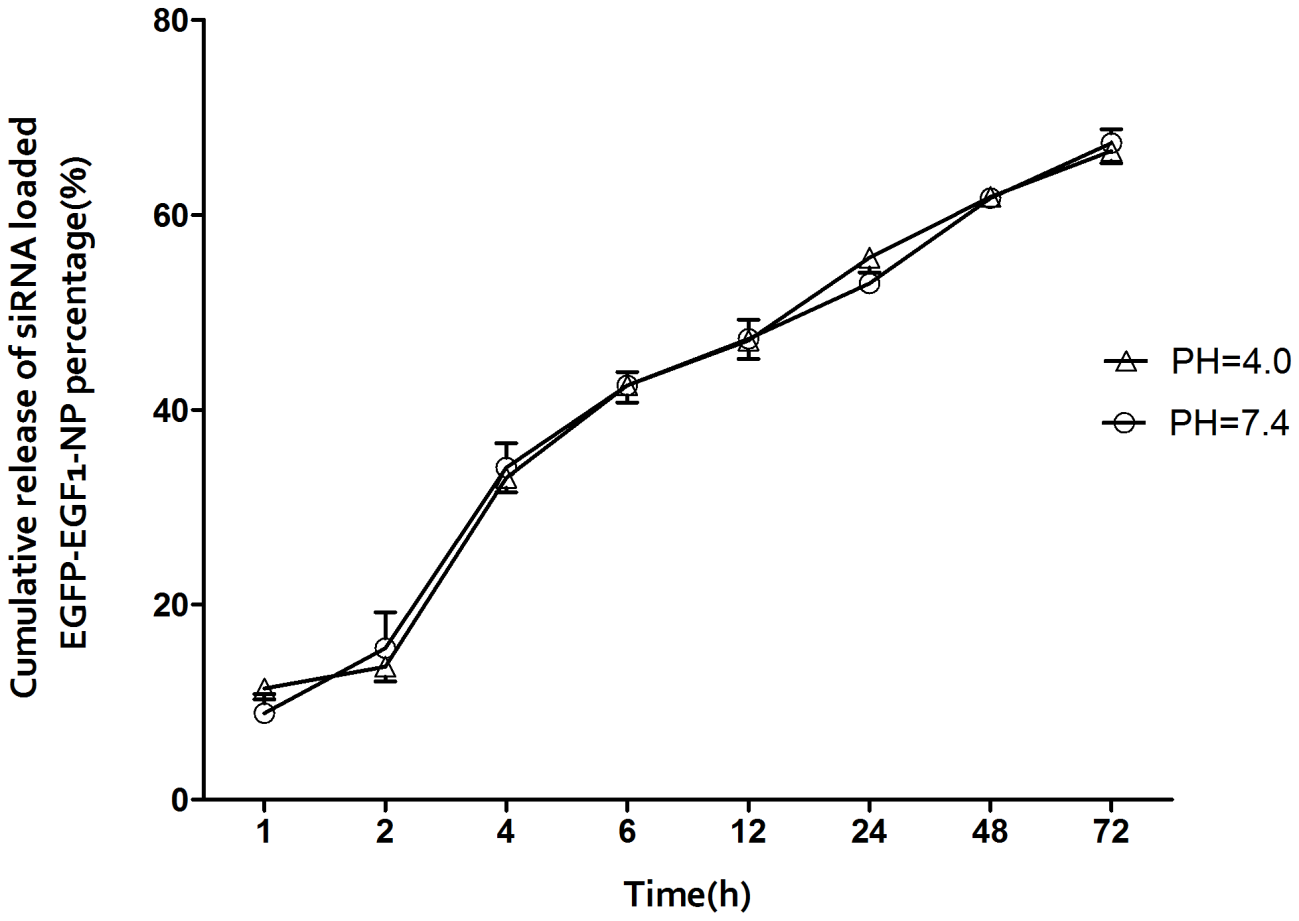
The siRNA-loaded ENPs that were diluted in different pHs of PBS (0.01 M). The siRNAs that were extracted from the nanoparticles at different points in time were examined. The cumulative release rates were calculated for n = 3, as the mean±SD.

The cumulative release rates of siRNA in PBS (0.01 M) over 6 hours at pHs of 4.0 and 7.4 were 42.5% and 42.49%, respectively. The siRNAs were delay-released over the next 72 hours. There was no significant difference in the *in vitro* release rate (Fig. 2).

### 3.2. BMECs’ Uptake of Nanoparticles and siRNA Cellular Tracking

**Figure 3:**
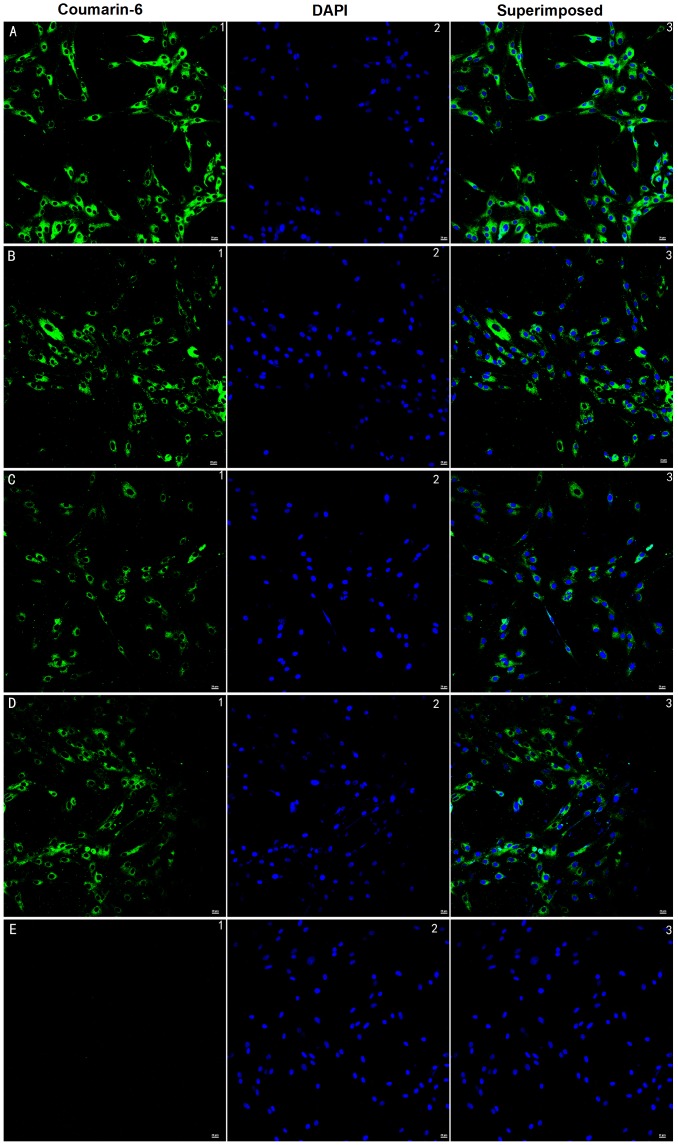
BMECs’ uptake of siRNA-loaded and 6-coumarin-loaded nanoparticles at 37°C over 4 h. (A) BMECs co-incubated with TNF-α (100 ng/ml) and ENPs. (B) BMECs co-incubated with TNF-α (100 ng/ml) and NPs. (C) BCECs incubated with just ENPs. (D) BMECs incubated with just NPs. (E) BMECs incubated with just PBS. Green: 6-coumarin labeled nanoparticles. The cells nuclei were examined by fluorescent microscopy after staining with 1 µg/ml DAPI for 10 min at room temperature. Red: Cy3-labeled siRNAs. Blue: cell nuclei. The bars are 20 µm.

**Figure 4:**
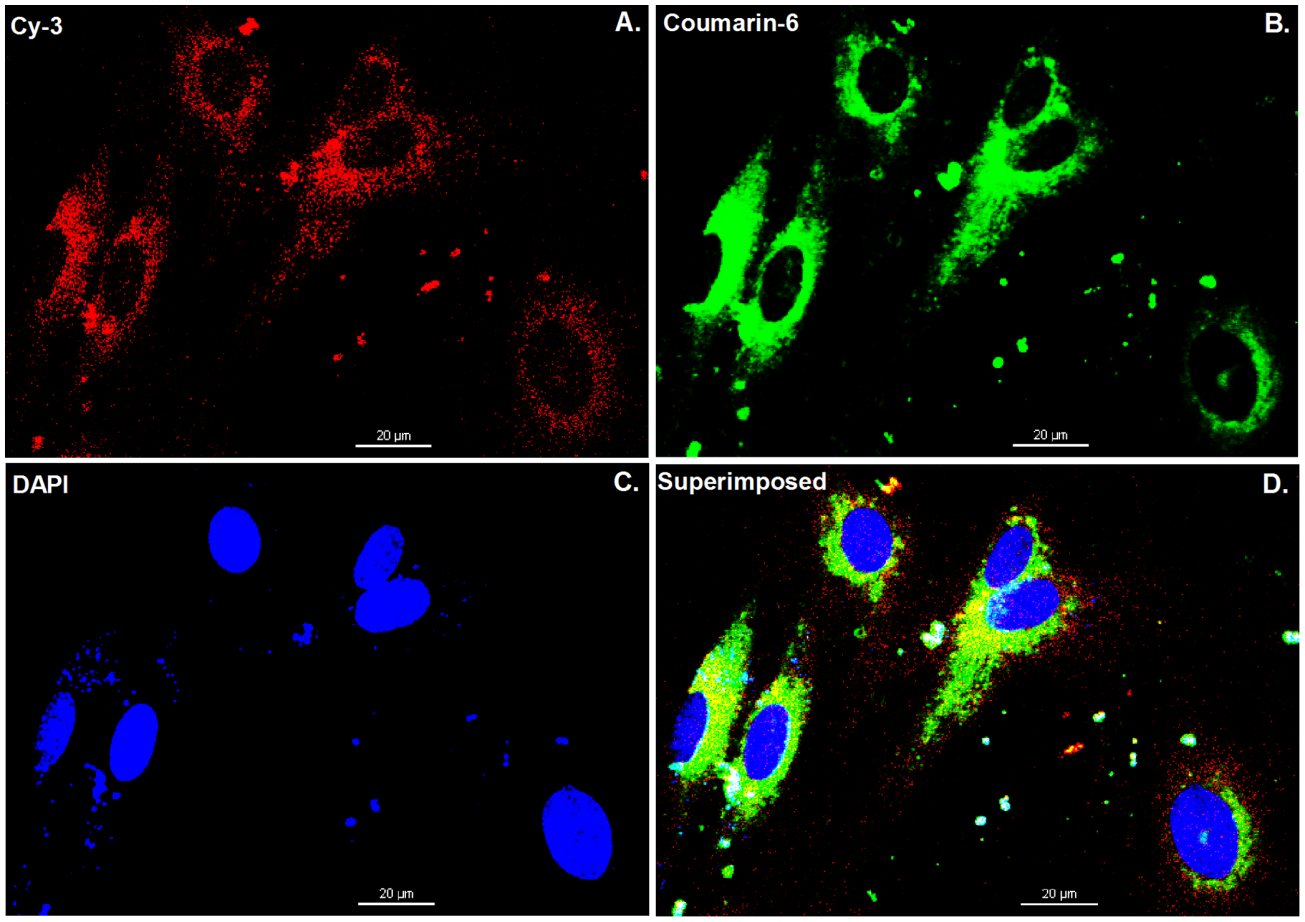
Intracellular localization of Cy3-labeled siRNAs and 6-coumarin-loaded ENPs. The cells were cultured in 35 mm glass bottom dishes for 24 h, then co-incubation with ENPs and TNF-α (100 ng/ml) at 37°C for 4 h, and subsequently examined by confocal microscopy. Red: Cy3-labeled siRNAs (A). Green: 6-coumarin labeled nanoparticles (B). Blue: cell nuclei (C). Yellow: superimpose red fluorescence on green fluorescence (D). After incubated with 6-coumarin labeled ENPs for 4 h, many of the ENPs had been phagocytize by cells and released Cy3-labeled siRNAs. The bars are 20 µm.

The BMECs could take up NPs and ENPs, both the 6-coumarin-labeled and siRNA-loaded ones (Fig. 3.C and D). The fluorescence intensity of the TNF-α-induced BMECs was higher for the ENPs than for the NPs (Fig. 3.A and B). As depicted in Fig. 4, the fluorescence intensity of the TNF-α-induced BMECs (Fig. 3.A) was higher than the normal ones (Fig. 3.C) when treated with the ENPs. This result illustrated that the ENPs could more efficiently bind to the TF-expressing BMECs than the NPs could. This conclusion was consistent with a previous study [19].

As depicted in Fig. 4, it was clear that the injured cells could up take siRNA-loaded ENPs (Fig. 4.B) and the Cy3-labeled siRNA could be transferred into the treated BMECs (Fig. 4.A) with 4 h using the ENPs. And there was almost coincidence between the 6-coumarin-loaded ENPs (Green) and the Cy3-labeled siRNAs (Red) in cytoplasm (Fig. 4.D).

### 3.3. Cytotoxicity Evaluation

**Figure 5:**
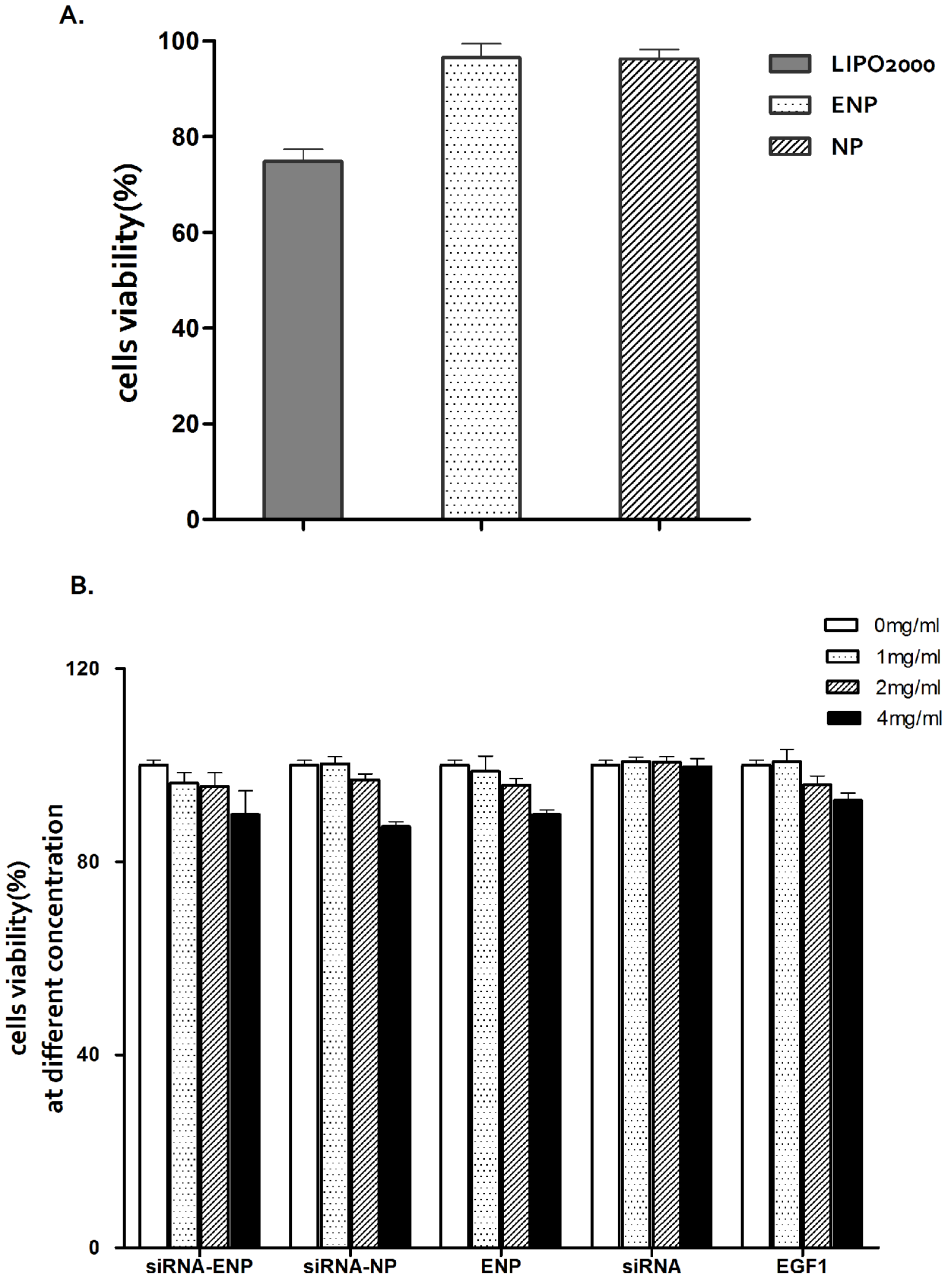
Cell viability assays. (A) BMECs were transfected with different nanoparticles and liposomes at 37°C for 24 h. (B) BMECs were treated with different concentrations of nanoparticles at 37°C for 24 h. The assays were performed in triplicate and the standard errors are shown.

Fig. 3 illustrates the cytotoxicities of different nanoparticle formulations evaluated using the CCK-8 kit assay. Our results showed that cells transfected with Lipofectamine 2000 exhibited a higher cytotoxicity over a 24 h time period than the cells transfected with ENPs or NPs which exhibited almost no cytotoxicity. In the primary BMECs transfected, the cell viability was 96.51±2.95% with ENP transfection and 96.28±2.02% with NP transfection as compared with only 74.82±2.57% with Lipofectamine 2000 transfection (Fig. 5.A). Moreover, there was no significant dose-dependent cytotoxicity for the different nanoparticles (Fig. 5.B).

### 3.4. Effect of TF-siRNA-loaded ENPs on TF Expression

**Figure 6:**
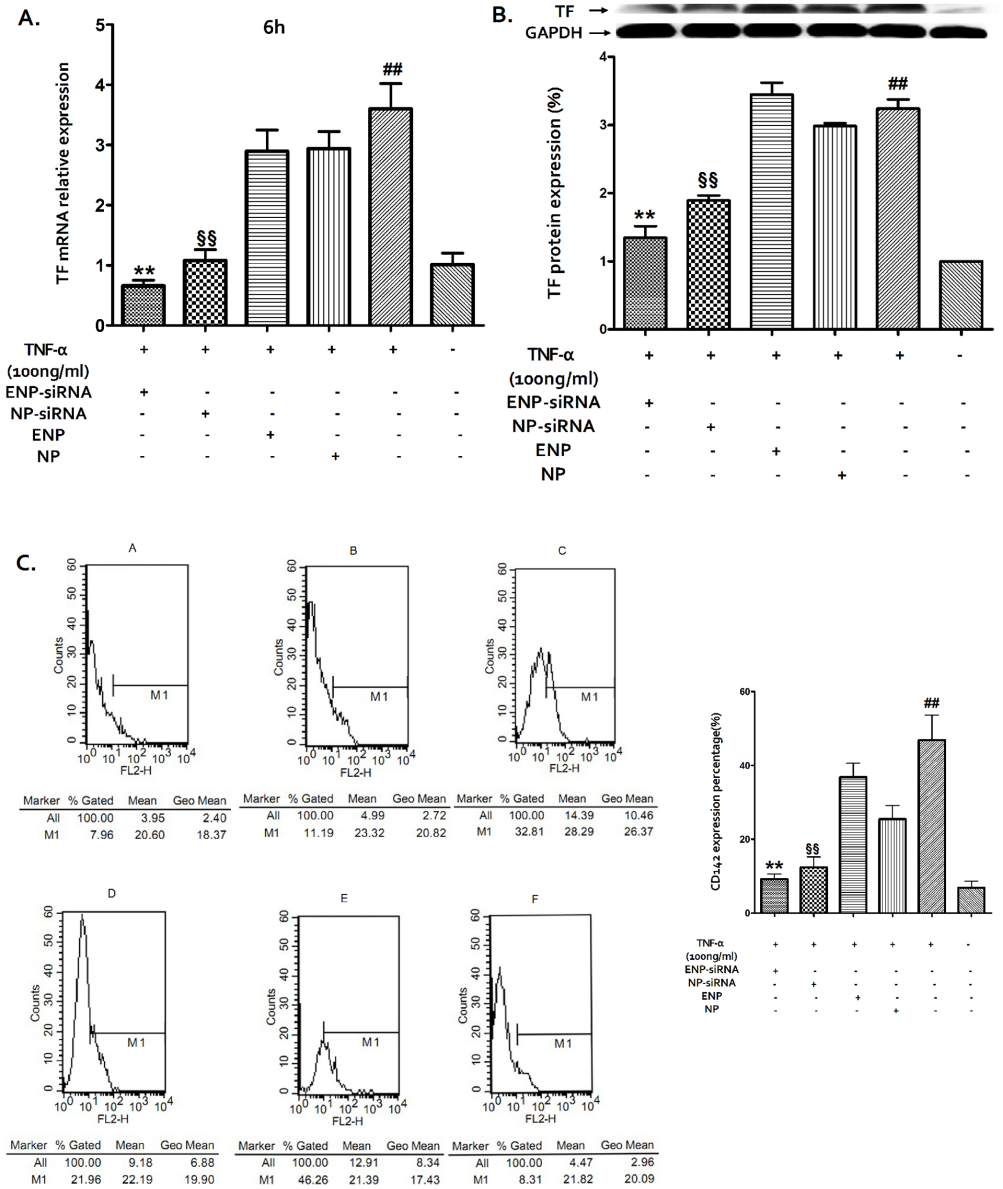
The TF expression was determined by real-time PCR (A), western-blot (B), and flow cytometry (C). The experiments were performed in triplicate, and the standard errors are shown. (A) The TF mRNA levels from the differently treated BMECs were normalized to the GAPDH mRNA level. The relative fold of the TF mRNA levels from the treated BMECs was normalized to the negative control from the normal BMECs. **P<0.01, §§P<0.05, ##P<0.05. (B) The relative fold of TF protein expression was normalized using GAPDH protein and normal BMECs. **P<0.05, §§P<0.05, ##P<0.05. (C) The percentages of CD142-expressing cells were normalized using normal BMECs. **P<0.05, §§P<0.05, ##P<0.05.

The real-time PCR results showed that the TF mRNA level of the injured BMECs exhibited an approximately 4.1-fold decrease following transfection with TF-siRNA-loaded ENPs compared with the control (Fig. 6.A). The downregulation efficiency is higher than the NP-based transfection rate.

The TF protein levels were determined by western blot (Fig. 6.B) and flow cytometry (Fig. 6.C). The western blot results showed that the TF protein expression was only 58.5% in the injured BMECs and that the downregulation efficiency exhibited a 1.41-fold increase compared with that for TF-siRNA-loaded NP transfection. The flow cytometry results confirmed the western blot results. The CD142 expression percentage in the injured BMECs was 9.17% following transfection with TF-siRNA-loaded ENPs, while the percentage in injured BMECs without transfection was 42.26%.

### 3.5. Effect of TF-siRNA Loaded ENPs on TF Activity

**Figure 7:**
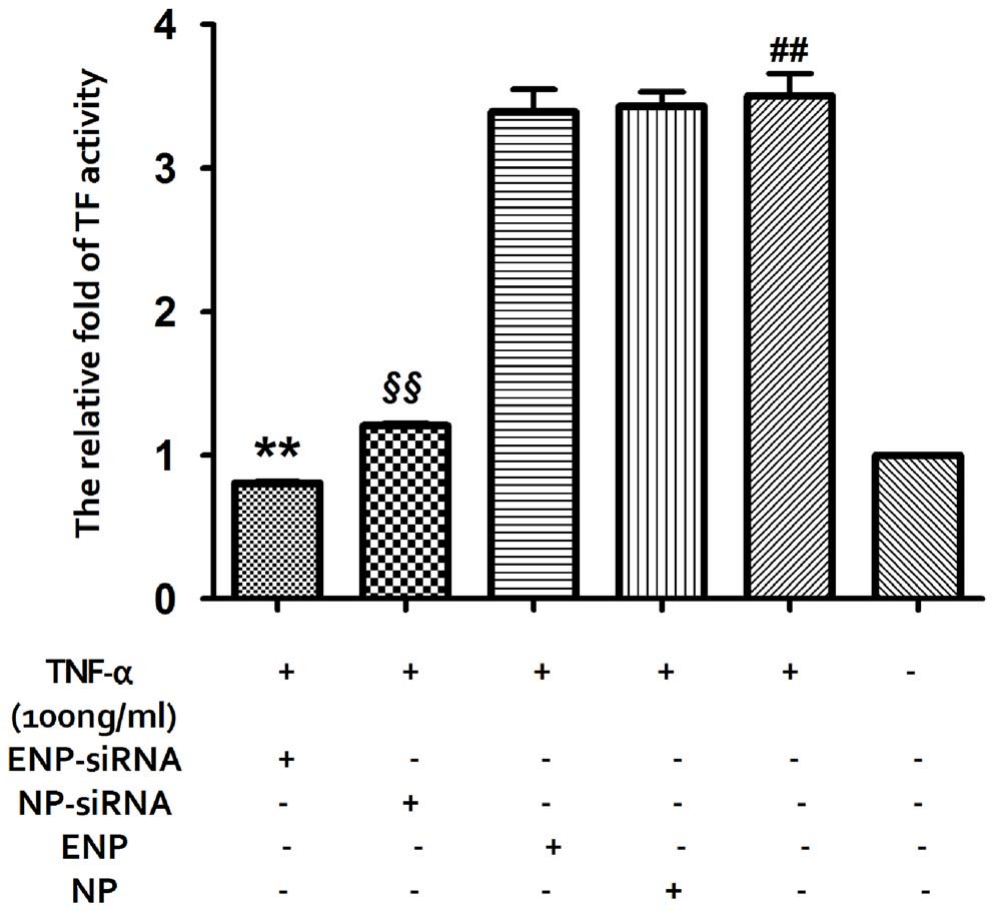
The TF activity was determined using the TF activity assay kit. The relative fold of TF activity was normalized using the normal BMECs. **P<0.01, §§P<0.01, ##P<0.05.

To evaluate the TF activity of the different BMECs treatments, TF activity assays were performed (Fig. 7). The results showed that the TF activity of the injured BMECs exhibited an approximately 3.52-fold compared with the normal cells. Following the TF-siRNA-loaded ENPs transfection, the TF activity was 19.59% that for the injured BMECs. This activity was a 1.5-fold increase over the TF-siRNA-loaded NPs transfection activity.

## Discussion

Tissue factor (TF) is a 47-kDa transmembrane cellular receptor for FVII/VIIa, which is no longer perceived as an ‘alternative’ coagulation factor, but rather as a central trigger for coagulation cascade, and an important cell-associated signaling receptor activated by factor VIIa and TF interacts with several other regulatory entities, such as protease-activated receptors (PAR-1 and PAR-2) [6], [7], [27]. Moreover, TF is involved in the pathogenesis of cancer, such as tumor growth, metastasis, angiogenesis, and, possibly, formation of the cancer stem cell niche [27]. Therefore, therapeutic strategies are being developed to specifically interfere with TF.

SiRNA has been generally investigated for use in therapies for various diseases. TF-specific siRNAs have been investigated in previous studies on breast cancer [28]. The use of siRNA is limited due to its poor stability, insufficient cellular uptake, and the safety problems associated with their carriers. An ideal delivery carrier for siRNA needs to have several features such as targeted delivery to specific cells or tissues, persistently activate RNA interference (RNAi) with a high efficiency, and an excellent safety profile [29]. PLGA nanoparticles are a suitable choice and can be modified for targeted delivery [30], [31]. Here, EGFP-EGF1 modified PLGA nanoparticles served as a new carrier for TF-siRNA. First, the nanoparticles loaded with siRNAs have an average diameter of approximately 100 nm, which is suitable for pharmaceutical applications [32]. To visualize the nanoparticles they were labeled with 6-coumarin. The cellular uptake assay shows that the normal primary BMECs could take up ENPs and NPs (Fig. 3.C and D), which are internalized in the cells through fluid-phase pinocytosis and endocytosis. However, the fluorescence intensity of the TNF-α-induced BMECs is higher for the ENPs than for the NPs (Fig. 3.A and B) which accounts for the TNF-α-induced BMECs could take up the ENPs better than the NPs. And the injured BMECs could take up the ENPs better than the normal cells (Fig. 3.A and C). The results show that EGFP-EGF1 could enhance the location concentration of PLGA nanoparticles in the injured BMECs because of its ability to target these TF-expressing cells [19]. As the *in vitro* release experiments show, siRNAs can be released from the nanoparticles (Fig. 2). Moreover, the Cy3-labeled-siRNAs (Red) and the 6-coumarin labeled ENPs (Green) can be determined in the injured BMECs at the same time after transfection (Fig. 4.D). It illustrates the released siRNAs can enter the injured primary BMECs by the ENPs. All of these results show that the ENPs have the capacity for targeted delivery to specific cells. Second, when the TF-siRNA was transfected into the injured BMECs by the ENPs or NPs, it guided sequence-specific gene silencing of the target mRNAs that they were perfectly complemented by directing the RNA-induced silencing complex (RISC) to mediate site-specific cleavage and to destroy the mRNA [33]. The results were shown in the mRNA and protein levels we can see an efficient downregulation of TF in the injured BMECs. However, the gene knockdown efficiency is apparently different for the treatments using the siRNA/ENPs and the siRNA/NPs. It’s possible that more of the TF-siRNA carried by the ENPs could enter the injured BMECs than that carried by the NPs because the new carrier has the targeted delivery. The efficient gene silencing shows that the new carrier has the capacity for persistently activate RNAi with a high efficiency. The use of traditional carriers, such as liposomes and viral vectors, is limited by safety issues, such acute toxicity, cellular immune response, and quality control. However, PLGA is approved by the Food and Drug Administration (FDA) and European Medicines Agency (EMA) for use in various drug delivery systems in humans. As the CCK8-assay showed, the EGFP-EGF1-PLGA nanoparticles had no significant dose-dependent cytotoxicity over 0–4 mg/ml range, while the Lipofectamine2000 had apparent dose-dependent cytotoxicity (Fig. 5). Thus, the new carrier has an excellent safety profile.

It is generally believed that most TF on the surfaces of cells surrounding blood vessels exist in a state with very little procoagulant activity and must undergo a transformation to become fully active [34]. The aberrant activation of TF-mediated coagulation leads to intravascular thrombus formation. It is also well established that active TF is a link between the activation of the coagulation system and cancer [27]. As the TF activity assay showed, the TF activity of the injured primary BMECs is approximately 4 times higher than that of the normal ones. Nevertheless, there is a significant decrease following ENP-mediated siRNA transfection (Fig. 7). Perhaps the efficiency decrease in TF expression has an effect on the TF activity.

Endothelial injury is involved in the pathogenesis of pathologic processes ranging from vascular diseases to cancer and metastasis [35]. TNF-α mediated endothelial injuries are related to acute inflammation, which leads to TF overexpression [36]–[37]. BMECs are a type of endothelial cell and play an important role in many brain diseases. Therapies targeted to injured endothelium are in urgent demand. In this study, we have proven that ENPs could target these cells and efficiently transfer TF-siRNAs into them. Then, the siRNAs would bring about gene knockdown. Thus, this new targeted delivery system for TF-siRNAs may be used for brain diseases that involve endothelium injury.

In this study, the ENPs that served as a new targeted delivery system of siRNAs have been shown to target injured BMECs. Western blot and flow cytometry analysis results revealed that ENP-mediated siRNA transfection is more efficient and longer sustained than NP-mediated transfection in injured primary BMECs. Functional assays (TF activity assays) confirmed that the TF-siRNAs released from the nanoparticles maintained their functional integrity. The new delivery system showed almost no cytotoxicity. Our findings suggest that the use of these ENPs for downregulation of TF expression may serve as an effective treatment for various diseases that involved endothelium injury. Our future studies will focus on the use of these ENPs to treat these diseases *in vivo*.
