# Correction: EGFP-EGF1-Conjugated PLGA Nanoparticles for Targeted Delivery of siRNA into Injured Brain Microvascular Endothelial Cells for Efficient RNA Interference

**DOI:** 10.1371/journal.pone.0303121

**Published:** 2024-05-01

**Authors:** Chen Chen, Heng Mei, Wei Shi, Jun Deng, Bo Zhang, Tao Guo, Huafang Wang, Yu Hu

After this article [1] was published it was noted that Fig 1D and 1E showed overlapping fields of the same data at slightly different magnifications. In the corrected Fig 1 provided here, panel D has been replaced with the correct data from the original experiment. The original underlying data files for panels D and E are in S1 and S2 Files, respectively.

**Fig 1:**
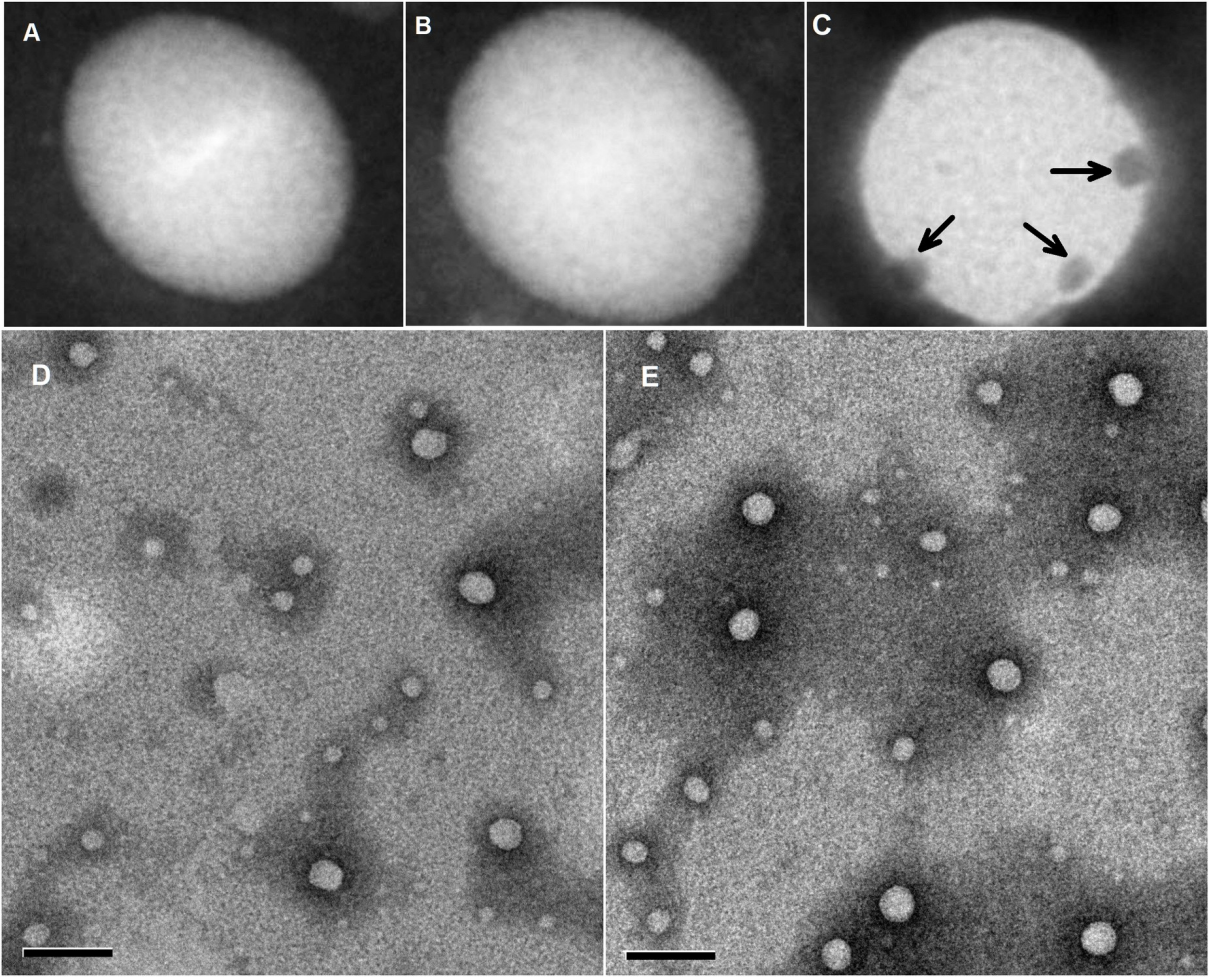
The different nanoparticles that were negatively stained with a 1% phosphotungstic acid solution. (A) siRNA-loaded NPs stained with EGF primary antibody and with 10 nM of colloidal gold-labeled rabbit anti-goat IgG. (B) siRNA-loaded ENPs stained with 10 nM of colloidal gold-labeled rabbit anti-goat IgG. (C) siRNA-loaded ENPs stained with EGF primary antibody and stained with 10 nM of colloidal gold-labeled rabbit anti-goat IgG. The arrows point to the fusion protein EGF1, which conjugated to the surfaces of the nanoparticles. (D) siRNA-loaded NPs. (E) siRNA-loaded ENPs. The bars shown in (D) and (E) are 200 nm.

## Supporting information

S1 File Original data underlying Fig 1D. (JPG)

S2 File Original data underlying Fig 1E. (JPG)
