# Supplementary figures and images for: Correction: EGFP-EGF1-Conjugated PLGA Nanoparticles for Targeted Delivery of siRNA into Injured Brain Microvascular Endothelial Cells for Efficient RNA Interference
Source: PLoS One. 2024 May 1;19(5):e0303121. doi: 10.1371/journal.pone.0303121 (PMC11062518; doi:10.1371/journal.pone.0303121)

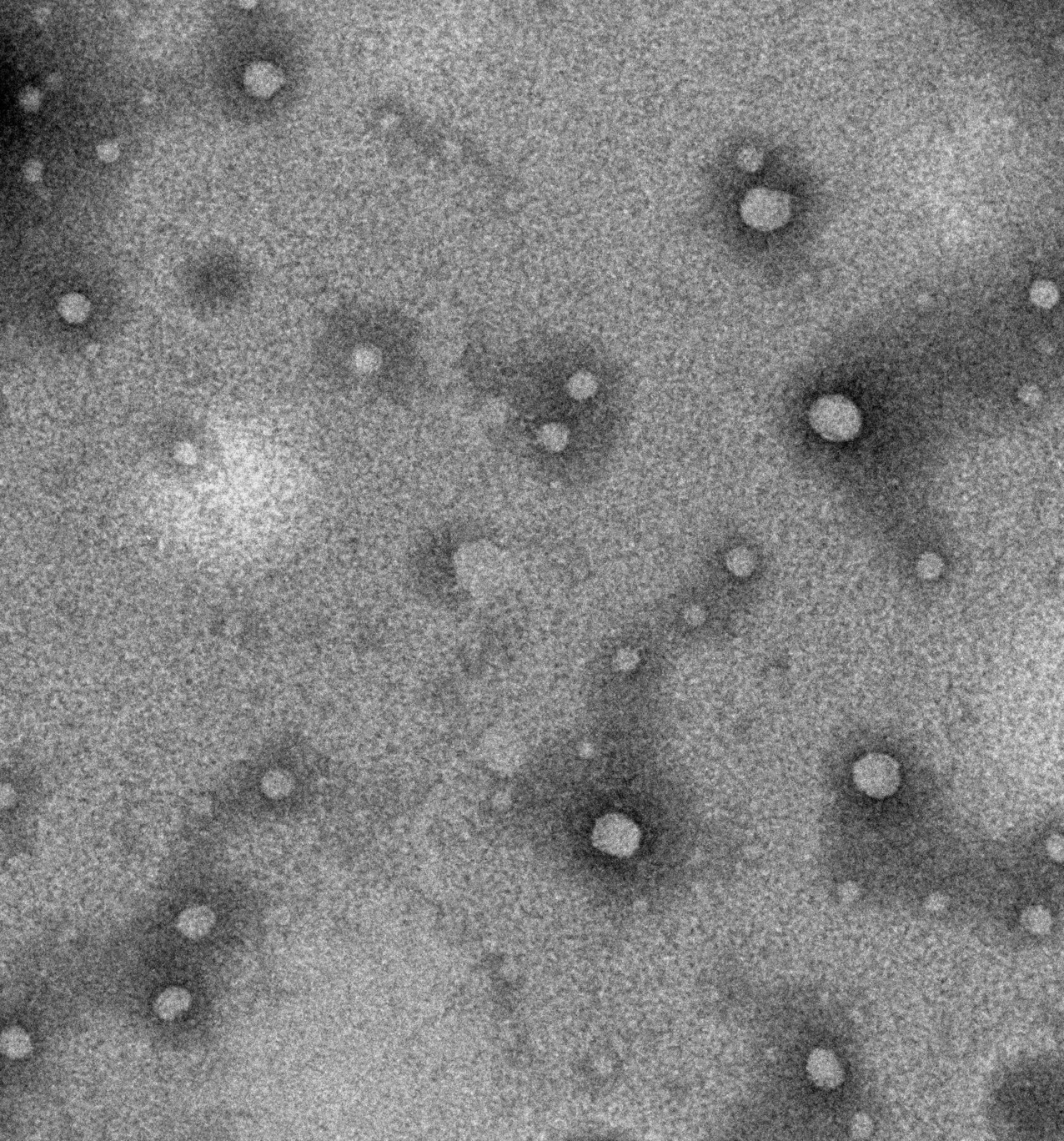

Supplement: S1 File — (JPG) [file pone.0303121.s001.jpg]

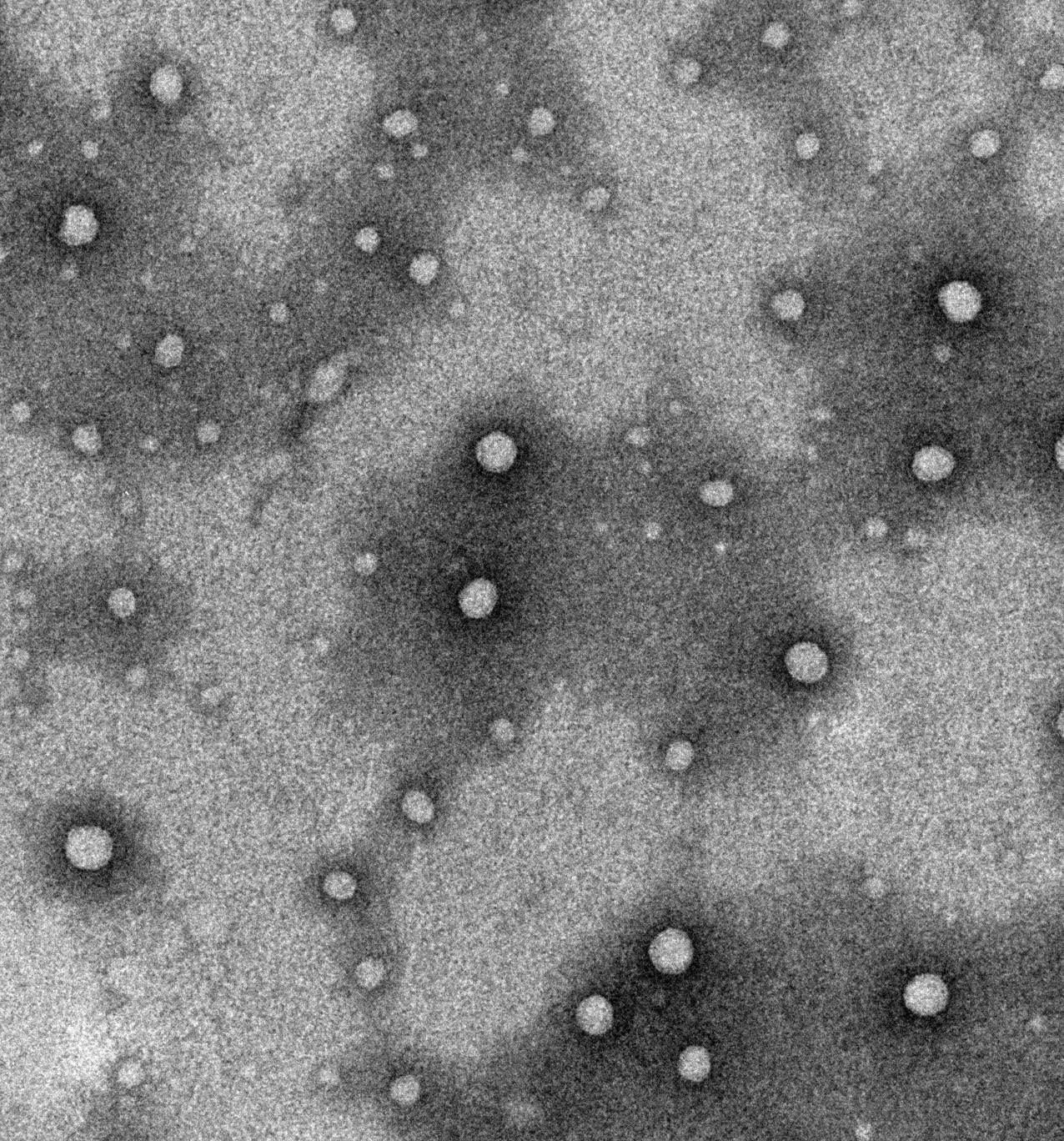

Supplement: S2 File — (JPG) [file pone.0303121.s002.jpg]
